# Class-Room Talks, No. 6

**Published:** 1888-07

**Authors:** J. T. Kent


### CLASS-ROOM TALKS. No. 6.

(From Lectures by Professor J. T. Kent, M. D.)

The hygeists of our city have made many and violent efforts to prevent the spread of diphtheria with this result: the more they fumigate, notify, and placard, the worse matters seem to get. I find it hard to prevent their entrance within my patient's door with their many and odoriferous accompaniments. These logical city fathers are always on the rampage; always hunting after germs; always killing germs; it would seem there ought not to be a germ left alive in the world, they have been so vigorously hunted, and yet disease still stalks abroad *unchecked.*

Let me tell you something. Just so soon as you find the homœopathic remedy for diphtheria, just that soon have you killed infection, and the *room* and the *patient* are *free* from it. In small-pox, scarlet fever, etc., such diseases as may be carried by fomites, they may still be spread by the fomites before infected, and yet the infection from the person be dead; the great manufactory of the poison is stopped. Therefore, the disinfecting matters and methods only prevent the action of the remedies and injure your patient's chances for recovery.

No greater mistake can be made than to combat so grave a zymosis as diphtheria by the external use of medicines.

There are three great reasons for opposing local treatment: First, it does not benefit the patient, it is only removing a *result* of disease and not reaching in any manner the zymosis; second, the true index or guide to the correct treatment is removed, changed, or obscured by such a management; third, the confidence in remedies is very strong with physicians who do *not*, and very *feeble* in all who *do*, use local treatment.

By this we must conclude that lack of success with the combined means has led to lack of confidence, and that *success*, following the constitutional treatment, has established the confidence that every correct prescriber carries with him to the bedside of this apparently grave disease.

The disinfectants and germicides are not without a place, but more attention has been given to germicides than to the materia medica. The confessions in our societies are evidence of the lack of the study of the remedies belonging to each individual case, and the generalization and treatment of the disease instead of the patient will account for the failures in practice. To fix upon a set of remedies, of which we would say, these are medicines for diphtheria, is *not* in harmony with homœopathic prescribing, yet it is but too common in this advanced day of medical knowledge.

The remedy that will speedily cure each and every case *can* be selected, and is often a remedy never before named for the disease. When Ignat., Cham., Bry. have been known to cure diphtheria that has exhibited a profusion of typical pseudo-membrane, who can say what remedy may not cure the disease? Who, on the other hand, would select one of these remedies without the most careful individualization and the most positive reasoning? Thus why select any remedy without the most positive reasons?

Our principles abound in reasons for action, but "when in doubt, wait." We do not say wait a week, nor a day, but wait and watch. An hour or two hours will be well spent in watching the symptoms so that a positive prescription can be made. We think some one of you may say, "The parents might get anxious." We answer, if the physician cannot command the respect and confidence of his families there are plenty of vacancies in other vocations. Nothing can be offered as a substitute for a correct prescription in such a grave disease. To accomplish this, the patient must be examined the more thoroughly until every doubt about the similarity has been removed.

The patient never grows worse after he has taken the first dose of the appropriate remedy; he may remain stationary for thirty-six hours in a very grave case, but he cannot grow worse. Generally, he feels better in twelve hours, and the membrane begins to decrease in twenty-four hours in the reverse order of its coming, and in very severe cases has *all* disappeared in six days.

Lach. or Merc-cy. may have cured a case in a given family and not cure another case in the whole season. These are not remedies for diphtheria, except when indicated by the symptoms.

This season just passed has furnished a great scare because of some painful cases of follicular tonsilitis, which Bell. has proved able to cure in most instances. The city has been whitewashed by our Health Department almost indiscriminately, but that it has accomplished much there is room for grave doubt.

The few cases of diphtheria have been North and South, while a central belt seems almost entirely to have escaped. The escape has *not* been due to fumigation.

Of the few deaths, inappropriate treatment has been the cause, also a cause of the great scare.

You can see we have no fixed treatment for diphtheria, but we have a fixed treatment for all suffering patients, suffering from no matter what constitutional wrongs, whether epidemic, endemic, or sporadic, infection or contagion, that treatment is, if successful, homœopathic.

To make use of this treatment, the physician must know *two* grand things, viz.: the materia medica and the principles that govern its application.

To furnish a treatise upon diphtheria would be impossible, as the whole range of the materia medica and the philosophy must be commanded, so that every turn of affairs, when the question arises of "what is to be done?" the principles must settle the use to be made of the well-selected remedy; these principles are never faulty when you know them, it is only when the principles are not known that they refuse to be a help at the bedside. S. L. G. L.

